# Frontiers and Structural Engineering for Building Flexible Zinc–Air Batteries

**DOI:** 10.1002/advs.202103954

**Published:** 2021-12-22

**Authors:** Tao Zhang, Ningxiang Wu, Yanhua Zhao, Xinglong Zhang, Jiansheng Wu, Jiena Weng, Sheng Li, Fengwei Huo, Wei Huang

**Affiliations:** ^1^ Key Laboratory of Flexible Electronics Institute of Advanced Materials Nanjing Tech University Nanjing 211816 China; ^2^ Frontiers Science Center for Flexible Electronics Xi'an Institute of Flexible Electronics (IFE) Xi'an Institute of Biomedical Materials & Engineering Northwestern Polytechnical University 127 West Youyi Road Xi'an 710072 China; ^3^ State Key Laboratory of Organic Electronics and Information Displays & Jiangsu Key Laboratory for Biosensors Institute of Advanced Materials Nanjing University of Posts and Telecommunications Nanjing 210023 China

**Keywords:** battery structure, flexible electrodes, flexible electronics, solid‐state electrolyte, Zn–air batteries

## Abstract

With the development of flexible devices, the demand for wearable power sources has increased and gradually become imperative. Zinc–air batteries (ZABs) have attracted lots of research interest due to their high theoretical energy density and excellent safety properties, which can meet the wearable energy supply requirements. Here, the flexibility of energy storage devices is discussed first, followed by the chemistries and development of flexible ZABs. The design of flexible electrodes, the properties of solid‐state electrolytes (SSEs), and the construction of deformable structures are discussed in depth. The researchers working on flexible energy storage devices will benefit from the work.

## Graphical abstract

The flexible Zn–air batteries (ZABs) are considered one of the most promising flexible energy supplies due to the advances in energy density and safety properties. Here, the design of electrodes, solid‐state electrolytes, and deformable structures for flexible ZABs are discussed.

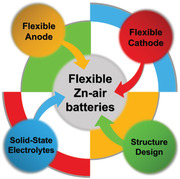

## 1 Introduction

Electronic technology and material science have promoted the emergence of flexible electronics, especially wearable smart devices.^[^ ^1^, ^2^ ^]^ Compared with traditional portable electronic equipment, wearable electronics are usually operated under deformable conditions such as bending, twisting, or even stretching.^[^ ^3^, ^4^, ^5^ ^]^ These have inevitably raised new requirements for flexible energy storage devices.^[^ ^6^, ^7^, ^8^ ^]^ Rechargeable battery is the most popular and promising technology in this area due to its high energy density, high power density, long cycle life, and technology maturity. Lithium‐ion batteries (LIBs) have been well developed in the last decades, becoming the first choice of wearable energy storage devices.^[^ ^9^, ^10^ ^]^ However, considering the chemical activity of lithium and the flammability of the organic electrolytes, there are still barriers to practical applications on flexible devices.^[^ ^11^, ^12^, ^13^ ^]^ Meanwhile, energy storage equipment that can achieve higher energy density with a long‐life energy supply has always been a goal pursued by researchers.^[^ ^14^, ^15^ ^]^ Therefore, developing flexible batteries beyond lithium with enhanced safety and higher energy density is essential.^[^ ^16^, ^17^, ^18^, ^19^, ^20^, ^21^ ^]^

In recent years, significant efforts have been devoted to designing new types of flexible batteries. According to these studies, some basic guidelines can be concluded to build an expected flexible battery: 1) the safety requirements must be the top priority for a flexible wearable battery. Highly active metals such as lithium or sodium will react violently and release a lot of heat when exposed to air.^[^ ^22^, ^23^ ^]^ Besides, using organic electrolytes will give a chance to thermorunaway, resulting in fire or even severe exploration when a short circuit or overcharge occurs.^[^ ^24^ ^]^ Thus aqueous electrolytes or solid‐state electrolytes (SSEs) with low flammability and metal anode with appropriate chemical activity (such as Zn) are preferred. 2) High energy density can guarantee a prolonged service time, making intelligent wearable devices with complex functions possible.^[^ ^25^ ^]^ In addition, the electrochemical cycling life is also essential.^[^ ^26^, ^27^ ^]^ 3) The flexible batteries should meet the requirements of mechanical stability under deformation conditions, such as continuously twisting, bending, or stretching.^[^ ^28^ ^]^ Besides, in some cases, wearing comfort is also an important factor to be taken into consideration when designing flexible batteries.^[^ ^29^ ^]^

**Figure 1:**
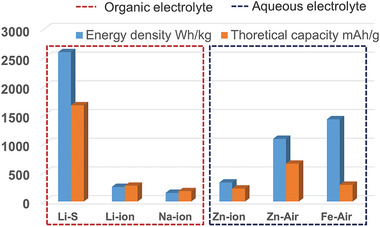
Theoretical energy density and theoretical capacity of several batteries. Data of LIB are calculated based on lithium cobalt oxide, and data of SIB is calculated based on sodium vanadium phosphate.

Beyond LIBs, many other electrochemical power sources also present good deformability through unique designs, such as supercapacitors, Na‐ion batteries, Zn‐ion batteries, lithium–sulfur batteries, and metal–air batteries.^[^ ^30^, ^31^, ^32^ ^]^ The theoretical energy density and the specific capacity of these batteries are summarized and compared in **Figure** **1**.^[^ ^33^, ^34^, ^35^ ^]^ Among all these batteries, Zinc–air batteries (ZABs) are pretty attractive due to their unique advantages. First, ZAB possesses excellent safety, as it is an aqueous‐based energy storage device.^[^ ^36^, ^37^, ^38^ ^]^ The appropriate chemical activity of Zn metal ensures the continuous electrochemical reaction without violent reaction in the air, regardless of the aqueous or solid‐state electrolytes. It can be suitable for flexible and wearable devices since it will avoid severe safety issues when the components are exposed to the air or short‐circuit, caused by continuously deformable actions. In addition to safety, ZABs also have advantages in energy density, which can guarantee the flexible device can be powered up for a longer time without charging.^[^ ^39^, ^40^, ^41^, ^42^ ^]^ Besides, considering the abundant earth crust reserves and the relatively low cost, ZABs can be one of the most promising choices for flexible energy storage applications.^[^ ^43^ ^]^ Recently, a series of excellent reviews have discussed ZABs from different aspects.^[^ ^44^, ^45^, ^46^, ^47^ ^]^ Here, we will focus on the device flexibility of ZABs from the design of the electrodes, the development of SSEs, and the architectures of the batteries, to summarize the recent advancements in flexible ZABs.

## 2 Zn–Air Batteries

Similar to Zn‐ion batteries, ZABs are also developed based on Zn–Mn batteries.^[^ ^48^, ^49^, ^50^, ^51^, ^52^ ^]^ At the end of the 19th century, the researchers found that oxygen could impact the capacity of the Zn–Mn battery. After that, alkaline electrolytes and catalysts were developed (such as the platinum carbon catalyst), and the basic structure of ZABs has appeared.^[^ ^53^, ^54^, ^55^ ^]^ Such kind of ZAB is still in its infancy with limited energy and power densities, often used in long‐term and low‐speed applications such as railway signal lights.^[^ ^52^, ^56^, ^57^ ^]^ Subsequently, the development of fuel cell technologies brought high‐efficiency catalysts and thus coin‐cell type ZABs. These inventions can release stable current and show excellent safety as primary batteries, leading to large‐scale applications for hearing aids and pagers.^[^ ^58^, ^59^, ^60^ ^]^ However, the further development of the rechargeable ZABs was overwhelmed by the successful commercialization of LIBs. In recent years, rechargeable ZABs have regained the researchers’ attention as new challenges and requirements emerge in the energy storage field.

**Figure 2:**
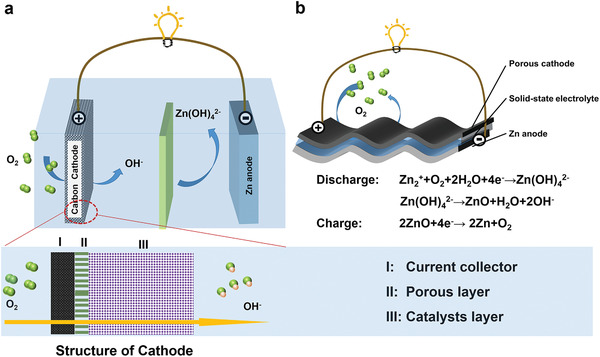
Schematic illustration of ZABs, and the cycling processes. a) Schematic illustration of ZABs and detailed structure of the cathode. b) Schematic illustration of flexible ZABs.

Functionally, a ZAB comprises a porous air cathode, a Zn metal anode, an electrolyte, a separator, and current collectors. The typical structure is shown in **Figure** **2**a.^[^ ^61^, ^62^ ^]^ The cycle of ZABs is generally divided into oxygen reduction reaction (ORR) and oxygen evolution reaction (OER) processes. During the discharge process, O_2_ will first be adsorbed on the surface of the solid catalyst, and then O = O will be activated and broken to generate the ORR intermediates. These intermediates will subsequently combine with Zn^2+^, forming discharge products (such as Zn(OH)_2_ or ZnO_2_) on the cathode. Ideally, during the following charge process, the deposited discharge products will be decomposed under the charge potential and release O_2_.^[^ ^63^, ^64^, ^65^ ^]^ However, the following issues need to be addressed to achieve the high efficiency and stable cycling of ZABs. The slow mass transfer caused by the three‐phase interface reaction, the poor conductivity of the discharge product, and the high energy barrier of the electrochemical reactions will all lead to a low cycle efficiency.^[^ ^66^, ^67^, ^68^ ^]^ Moreover, the corrosion, swelling, and irreversible dendrite growth of Zn metal will affect the cycle life.^[^ ^69^, ^70^ ^]^ After decades of exploration, considerable progress has been made to improve its performance by designing high‐efficiency bifunctional catalysts, expanding new electrolyte systems, and developing Zn anode protection methods.

Due to the advances mentioned above for safety and energy density, building flexible ZABs to power wearable intelligent electronics has attracted much research interest.^[^ ^71^, ^72^ ^]^ Currently, flexible ZABs are restricted by the troubling issues of ZABs themselves and the immature technology for device deformability.^[^ ^73^, ^74^, ^75^, ^76^ ^]^ The overall battery properties come from each component, and the flexibility of these critical parts will be discussed in the next section.

## 3 Design of Flexible ZABs

Similarly, a typical flexible ZAB functionally comprises a porous cathode, an electrolyte, a Zn metal anode, and current collectors with deformable properties (Figure 2b). The choices of these component materials and the construction methods of the battery will essentially affect the flexibility and the energy density of the entire system.

### 3.1 Flexible Cathodes of ZABs

The cathode is generally considered the most critical part of ZABs, as both ORR and OER processes occur there.^[^ ^77^, ^78^, ^79^, ^80^, ^81^, ^82^ ^]^ Therefore, many works have focused on this electrode, including the cathode structure construction, high‐stable interface construction, and the development of the catalysts.^[^ ^83^ ^]^ Functionally, the cathode of a ZAB is composed of the current collector, the air diffusion layer, and the catalysts.^[^ ^84^ ^]^ These three parts respectively play the roles of charge transport, oxygen transfer, and reaction energy barriers lowering.^[^ ^85^, ^86^ ^]^ Proper hydrophilicity and hydrophobicity on different sides of cathodes are also crucial for battery stability.^[^ ^87^ ^]^ In a word, a qualified flexible cathode should have the following characteristics: 1) excellent conductivity for prompted charge transfer; 2) proper porosity to ensure better O_2_ diffusion; 3) good bifunctional catalysis for advanced ORR and OER efficiency; 4) proper hydrophilicity and hydrophobicity for the formation of stable three‐phase interfaces. Besides the above considerations, flexibility must be taken into account as well.

#### 3.1.1 Substrate Materials of Cathodes

The substrate materials and construction methods largely determine the deformability of the flexible cathodes, and thus the traditional rigid substrates are no longer suitable.^[^ ^88^, ^89^ ^]^ Commonly used substrate materials for flexible cathodes include carbon‐based, metal‐based, and other kinds of materials. The electrical conductivity, deformability, and other properties of these substrate materials are summarized and listed in **Table** **1**. The potential materials are expected to facilitate gas diffusion, charge transfer, and flexibility simultaneously. Also, considering the long‐cycle demands, a suitable substrate material needs to be electrochemically stable for ZABs.^[^ ^90^, ^91^, ^92^ ^]^ Specifically, it is necessary to ensure that oxidation reactions do not occur to these substrates in an oxygen‐rich environment at 0–3 V (vs Zn metal). Considering high energy density requirements, it is necessary to keep the substrate as light as possible. Among these materials, carbon materials show good potential and have unique advantages in terms of conductivity, deformability, and air permeability. The other kinds of substrate materials often require more design when employed.

**Table 1:** Summary of some flexible cathode substrate materials in the literature

Type of substrate materials	Conductivity	Mechanical deformation and flexural strength of the substrate	The ability for gas diffusion	Stability in an electrochemical environment	Typical materials and Ref.
Carbon‐based materials	Excellent	Excellent	Good (normally with micro/meso pores)	Good (carbon corrosion)	Carbon cloth^[^ ^60^ ^]^ Carbon paper^[^ ^111^ ^]^ CNT^[^ ^104^ ^]^ Graphene^[^ ^146^ ^]^
Metal materials	Excellent	Good	Good (normally with macropores)	Good (metal corrosion or oxidation)	Nickel foam^[^ ^119^ ^]^ Stainless steel^[^ ^120^ ^]^ Cu foil^[^ ^199^ ^]^
Other materials	Normal	Normal	Normal (normally with micro/meso pores)	Normal (structural collapse)	COFs^[^ ^126^, ^127^, ^128^, ^129^ ^]^

Carbon‐based materials, such as carbon paper, carbon cloth, and carbon nanotubes, have been widely used in metal–air batteries due to their lightweight, excellent conductivity, and good chemical stability.^[^ ^93^, ^94^, ^95^ ^]^ A flexible 2D carbon substrate (e.g., carbon cloth) could be made of 1D carbon fibers through weaving or direct accumulation.^[^ ^96^ ^]^ Such a crosslinked structure shows good flexibility and has enough porosity to guarantee fast charge transfer.^[^ ^97^, ^98^ ^]^ In addition, the hydrophilicity and hydrophobicity of the carbon materials are adjustable through the regulation of the oxygen‐containing groups.^[^ ^99^ ^]^

As a kind of 1D carbon material with excellent conductivity, CNTs have been widely explored as flexible cathode materials.^[^ ^100^, ^101^, ^102^, ^103^ ^]^ Zhang and co‐workers designed a porphyrin‐based covalent organic framework (COF) on the CNTs as the support to form a self‐standing cathode.^[^ ^104^ ^]^ The manufactured porphyrin COF@CNT hybrid material exhibited impressive performance in flexible all‐solid‐state ZABs. The flexible ZABs with a COF@CNT cathode displayed a small voltage gap of 0.71 V and excellent stability (running for 200 cycles, and working stably under bending 90° and 180°). Some other CNT‐based materials could also be used as flexible substrates. Li et al. got a crosslinked CNT aerogel through the chemical vapor deposition (CVD) method.^[^ ^105^ ^]^ The aerogel was uniformly modified with nitrogen‐doped cobalt oxide to obtain a self‐supporting flexible cathode. This electrode exhibited excellent conductivity in flexible ZABs and good catalytic activity and cycle stability.

**Figure 3:**
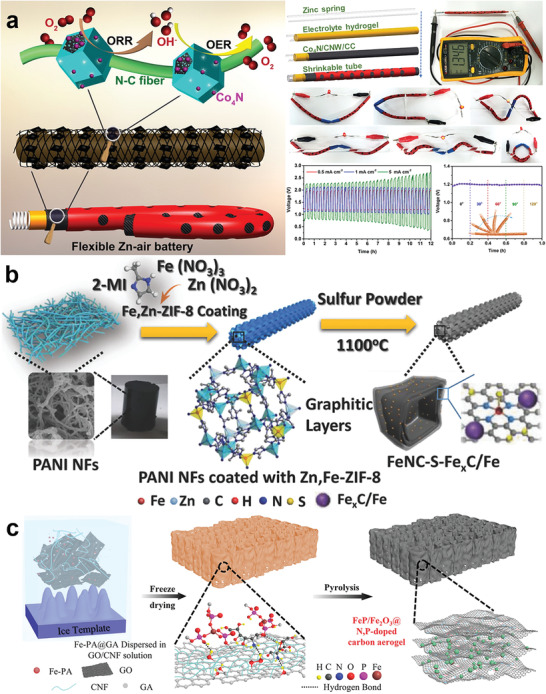
a) Scheme of the synthesis of Co_4_N/CNW/CC cathode and schematic illustration of the fabrication and structure characterization of the cable‐type Zn–air battery. Reproduced with permission.^[^ ^111^ ^]^ Copyright 2016, American Chemical Society. b) Synthesis scheme of the FeNC–S–Fe*_x_* C/Fe catalyst. Reproduced with permission.^[^ ^145^ ^]^ Copyright 2018, Wiley‐VCH. c) Schematic illustration of the preparation of the FeP/Fe_2_O_3_@NPCA cathode. Reproduced with permission.^[^ ^158^ ^]^ Copyright 2020, Wiley‐VCH.

In addition to CNTs, graphene and some other carbon materials have also been investigated as the skeleton to form substrate materials for flexible cathodes.^[^ ^106^, ^107^ ^]^ Liu and co‐workers coated the graphene sheets with a pre‐treated carbon cloth to create an interconnected and conductive network structure.^[^ ^108^ ^]^ Then, manganese oxides with hierarchical nanostructures were in situ grown in this large‐area carbon network. A cathode with uniform catalyst loading and good mechanical strength was formed as a result. It exhibited a lower polarization curve in flexible ZABs. This battery provided a power density of about 32 mW cm^−2^ and worked stably for 110 cycles. Notably, the battery showed a similar discharge/charge profile and good cycling performance after folding and unfolding 100 times. In other studies, the modified carbon cloth was also demonstrated to have excellent potential applications in the flexible cathode.^[^ ^109^, ^110^ ^]^ Zhang and co‐workers obtained the Co‐containing metal–organic framework (MOF) material in situ grown on the surface of the carbon cloth by hydrothermal method (**Figure** **3**a).^[^ ^111^ ^]^ The Co_4_N catalyst with high OER and ORR catalytic activity was obtained by pyrolysis of the MOF‐coated carbon cloth. Using this structure as a cathode, a 1D fibrous ZAB was constructed with a spring‐shaped Zn anode and a solid electrolyte. The battery exhibited a charge‐discharge overpotential of 1.09 V, a long cycle life, and excellent flexibility (almost no change in discharge performance at different bending angles).

Carbon‐based materials are acknowledged as one of the most widely used substrates in metal–air batteries. They have a wide range of sources, strong design ability, lightweight, hydrophobicity, and tunable surface affinity, which can meet the requirements of flexible ZABs well. With vast structure design possibilities, carbon‐based materials are competitively advantageous to the future flexible batteries design.

Metal materials are also often chosen as substrates for flexible cathodes due to their excellent electrical conductivity and high ductility.^[^ ^112^ ^]^ Mature metallurgical techniques and high ductility of metal ensure that flexibility can be obtained by simple processing.^[^ ^113^, ^114^ ^]^ After introducing the pores through corrosion or other methods, metal‐based materials could benefit mass transfer.^[^ ^115^, ^116^ ^]^ Notably, some metals possessing high ORR or OER catalytic activity themselves can be used directly as catalysts or catalyst precursors.^[^ ^117^, ^118^ ^]^ Sun et al. used nickel foam as the flexible substrate by introducing the cobalt precursor after the hydrothermal and calcination treatment.^[^ ^119^ ^]^ Nano‐Co_3_O_4_ arrays were thus grown on nickel foam, and the resulting Co_3−_ *_x_* Ni*_x_* O_4_/Co_3_O_4_ was used as a flexible cathode to form ZAB and Zn‐ion batteries. Both batteries showed good cycle stability, and the solid‐state ZAB based on this catalyst also has a certain degree of flexibility. In addition to nickel foam, many other metals are explored as the substrate material for flexible cathodes, such as stainless steel, copper foam, and cobalt.^[^ ^120^, ^121^, ^122^ ^]^ Most metal mesh substrates have considerable weight, usually leading to heavier electrodes and decreased battery energy density.^[^ ^123^, ^124^, ^125^ ^]^ Moreover, the porosity of these metal substrates is often not as good as that of carbon‐based materials. Worse still, some of the metal substrates are prone to cause undesired side reactions during cycling.

In addition to carbon and metals, other materials such as covalent organic polymers and MOF‐based composite materials have also been tried as substrates for flexible cathodes.^[^ ^126^ ^]^ COF is a porous material obtained by covalently connecting organic molecules. The designable pore structure is also beneficial to the flexibility of cathodes.^[^ ^127^, ^128^ ^]^ Zhang and co‐workers used a porphyrin‐based covalent organic framework with CNT scaffolds, obtaining a flexible cathode with bifunctional catalytic activity.^[^ ^104^ ^]^ The ZAB with this cathode demonstrated good electrochemical and flexibility performance (exhibited stable discharge and charging voltages of 1.22 and 1.98 V at 1.0 mA cm^−2^ at different angles). In addition, some composite materials such as carbon‐coated polymer frameworks have attracted much attention as well.^[^ ^30^, ^129^, ^130^, ^131^ ^]^ Among them, elastic polymers are typically used as the skeleton and coated with a carbon or metal shell to improve the stability and conductivity of the system. But the process sometimes requires ingenious or complex design.

#### 3.1.2 Preparation Methods of Flexible Cathodes

Apart from the substrate materials, the deformability and performance of the flexible cathode could also be affected by the preparation methods.^[^ ^132^ ^]^ According to the growth method of the catalyst layer, flexible cathodes can be simply divided into two categories, as shown in **Table** **2**. The catalyst layer is typically introduced through ex situ or in situ methods.^[^ ^133^, ^134^ ^]^ Constructing a flexible cathode via the ex situ method is technically the simplest and usually involves no additional equipment. High‐efficiency catalysis can be achieved through the introduction of a variety of catalysts.^[^ ^71^, ^135^, ^136^ ^]^ In contrast, the in situ method could keep the direct contact between the catalysts and substrates, promoting the charge transfer in the cathode.^[^ ^137^ ^]^

**Table 2:** Mechanical deformation of the flexible ZABs in the literature

Anode		Electrolyte	Cathode		Cell structures	Mechanical deformation of the batteries	Ref.
Material	Design approach		Material	Design approach			
Zn foil	0.25 mm	18 m KOH/PVA gel electrolyte	FeNC‐S‐Fe*_x_* C/Fe	Ex situ	2D Sandwich structure	180° bendable	[145]
Zn foil	0.1 mm	0.2 m ZnCl_2_ 18 m KOH/PVA gel electrolyte	CNT@POF	In situ	2D Sandwich structure	180° bendable	[104]
Zn spring	/	18 m KOH/PVA gel electrolyte	Co_4_N/CNW/CC	In situ	1D fiber shape	120° bendable/stretchable	[111]
Zn foil	0.1 mm	11.25 m KOH 0.25 m ZnO/PAA gel electrolyte	MnO*_x_*‐CC	In situ	2D Sandwich structure	Bendable	[60]
Zn foil	0.1 mm	0.2 m ZnCl_2_ 18 m KOH/PVA gel electrolyte	Co/N/O/GO	Ex situ	2D Sandwich structure	180° bendable	[146]
Zn particle	Electrospinning	6 m KOH PVA/PAA gel electrolyte	Co_3_O_4_	In situ	2D Sandwich structure	Bendable/100° twistable	[177]
Zn/Cu	Electrodeposition	KOH/PAA‐TEAOH gel electrolyte	Co_3_O_4_	Ex situ	2D Sandwich structure	90° bendable	[178]
Zn	/	KOH KI/PVA PAA GO based gel electrolyte	Pt/C	Ex situ	1D fiber shape/2D sandwich structure	2D bendable	[183]
Zn	Electroplating	6 m KOH/PANa cellulose and MBAA based gel electrolyte	/	Ex situ	1D fiber shape/ 2D sandwich structure	1D 500% 2D 800% stretchable	[185]
Zn powder	Coating	1 m KOH/cellulose membrane	Co_3_O_4_	Ex situ	2D sandwich structure	Bendable	[187]
Zn metal belt	Winding	0.1 m KOH/commercial gelatin based gel electrolyte	Fe/N/C	In situ	1D fiber shape	Bendable	[190]
Zn spring	/	6 m KOH 0.2 m Zn(Ac)_2_/PANa cellulose based gel electrolyte	Co–N–Cs	Ex situ	1D fiber shape	Stretchable/weavable	[191]
Zn foil		0.2 m Zn(Ac)_2_ 18 m KOH/PVA gel electrolyte	FeCo/Se‐CNT	In situ	2D sandwich structure	180° Bendable	[193]
Zn	Electrodeposited	KOH/PANa gel Electrolyte	Au foil	Ex situ	Wave structure 2D sandwich structure	300% Stretchable 85% Compressible	[196]
Interdigital Zn‐foil	/	KOH/PAM‐*co*‐PAA gel electrolyte	NdDCF‐OIM/Co‐800	Ex situ	Bridge‐island 2D sandwich structure	400% Stretchable	[199]

In the ex situ methods, a flexible cathode is mainly obtained by introducing the catalyst on the substrate via coating, printing, hot pressing, etc.^[^ ^138^ ^]^ The commonly used catalysts include precious metals, transition metals, and carbon‐based catalysts.^[^ ^139^, ^140^, ^141^ ^]^ Coating with binders can be the most used ex situ preparation method. A typical operation process of such a method is preparing and mixing the catalyst, polymer binder, and sometimes conductive agent into a slurry and then coating them on the substrate.^[^ ^142^ ^]^ The most common polymer binders are polytetrafluoroethylene (PTFE), polyvinylidene fluoride (PVDF), water‐soluble carboxymethyl cellulose (CMC), or agar.^[^ ^143^, ^144^ ^]^ Xu and co‐workers obtained a sulfur‐doped iron‐nitrogen co‐blended carbon catalyst by introducing sulfide atoms into the Fe–N–C catalyst, as shown in Figure 3b.^[^ ^145^ ^]^ Through the polymer binder, the catalyst was coated on the surface of carbon paper to form a flexible cathode. The battery delivered stable charging and discharging voltage while operating normally under 90° and 180° bending conditions. Some other methods could also be adapted to enhance the mechanical strength, such as hot pressing. Zhang et al. used intrinsic structural defects in nanocarbon to generate Co–N–O tri‐doped catalyst.^[^ ^146^ ^]^ The catalyst was coated on the carbon cloth with a polymer binder and hot‐pressed with a nickel frame to form a flexible cathode. Combined with PVA‐based gel electrolyte and Zn foil, a flexible ZAB was constructed and reported with good flexibility (stable cycling under 0°, 90°, 180° of bending) and electrochemical properties. In addition to coating and hot pressing, other efforts were devoted to preparing the catalysts as inks and then obtaining the flexible cathode by printing.^[^ ^147^, ^148^ ^]^

In general, it is relatively simple to obtain a flexible cathode through the ex situ methods, especially coating with polymer binders. But the use of the binder would have some negative impact on the flexible ZABs.^[^ ^149^, ^150^ ^]^ First, in terms of electrochemical performance, most of the binders do not have catalytic activity, and adding them would increase the weight of cathodes, lowering the system energy density.^[^ ^151^ ^]^ Moreover, the binder may cover the active sites of the catalyst and leads to low catalytic activity.^[^ ^152^, ^153^, ^154^ ^]^ Also, the powder catalysts coating on the flexible substrate would be easily immersed in the electrolyte, making it difficult to form a stable three‐phase interface. Furthermore, the decomposition or swelling of the polymer binder during the cycle may also cause catalyst failure, leading to battery performance decay.^[^ ^155^ ^]^ In addition, the flexibility of the cathode from the coating method is considered limited, as the coated layer would fall off in the cases of large‐scale bending, twisting, or stretching.^[^ ^156^ ^]^ Although the ex situ coating methods are universal and straightforward, they require choosing suitable polymer binders and controlling the adding amount. Besides, attention should be paid to the covered catalyst conditions when preparing the flexible cathode to avoid the low utilization efficiency of the catalyst.

The preparation of the flexible cathode can also be achieved by the in situ methods. Namely, the catalyst can be grown or deposited on a flexible substrate directly. It is generally accomplished through chemical synthesis or physical deposition (like hydrothermal synthesis, CVD, and physical vapor deposition (PVD)).^[^ ^146^, ^157^ ^]^ After preparation, a flexible cathode with catalytic activity can be obtained, promoting the charge transfer in the cathode.^[^ ^46^ ^]^ Lu and co‐workers used freeze‐casting and annealing methods to get the N, P co‐doping carbon aerogel as the substrate, as shown in Figure 3c.^[^ ^158^ ^]^ The carbon aerogel was then applied as a flexible substrate for in situ growing iron/iron oxide nanoparticles as a bifunctional catalyst. The obtained composites were used as cathode materials for flexible ZABs. The battery exhibited a specific capacity of 648 mAh g^−1^ at a current of 20 mA cm^−2^, and an energy density of 517 Wh kg^−1^ at 5 mA cm^−2^. The flexible ZABs can also power a LED bulb even when they were bent to 150°.

Besides the direct growth of catalysts, the precursor of the catalyst can also be in situ grown and then followed by carbonization or other processes for flexible cathodes.^[^ ^159^, ^160^ ^]^ Guo et al. chose the polyacrylonitrile (PAN) nanofibers as the substrate.^[^ ^161^ ^]^ The cobalt‐containing MOF‐coated nanofibers were obtained through in situ growth. The single‐atom cobalt decorated nitrogen‐doped carbon nanofibers were obtained through carbonization and acid washing. Combined with a PVA‐based solid electrolyte, a sandwich‐structured flexible ZAB was achieved. The obtained flexible ZAB could deliver good electrochemical performance under multiple deformations. Apart from these growth methods, flexible cathodes can also be obtained by in situ deposition methods such as atomic layer deposition (ALD) and PVD.^[^ ^162^, ^163^ ^]^

This in situ strategy could avoid the battery failure caused by polymer binders. It will keep sufficient contact between the catalyst and oxygen, which is hard to achieve in the powder catalysts.^[^ ^164^ ^]^ It will show better flexibility but is still affected and restricted mainly by the property of the substrate.^[^ ^165^, ^166^ ^]^ The flexible cathode obtained by this method can ensure the contact between the catalyst and the interface of each phase. However, a unique design of the cathode is needed if large‐scale deformation is required. Therefore, despite so many advantages, the limitations of the growth method would affect the in situ strategy.

After decades of development, the flexibility and catalytic activity of ZAB cathodes have significantly improved, although there are still many barriers restricting the design and applications of a flexible cathode.^[^ ^137^, ^167^ ^]^ Obtaining high‐quality large‐area flexible cathodes with satisfied electrochemical and mechanical performance is a current and constant pursuing target.

### 3.2 Flexibility of the ZABs Anode

Most research on flexible ZABs has focused on developing flexible air electrodes as both the ORR and OER reactions occur on the cathode. Nevertheless, it has to be admitted that the life of the Zn anode also essentially determines the ZAB cycle life, which is affected by the metal corrosion, swelling, and dendrite growth during the cycle.^[^ ^44^, ^168^, ^169^, ^170^ ^]^ A qualified anode should be equipped with the following characteristics: 1) It needs to form a stable solid electrolyte interphase (SEI) with the electrolyte, which could ensure the passage of sufficient Zn^2+^ while avoiding excessive corrosion of the Zn anode.^[^ ^171^, ^172^ ^]^ 2) It should possess adequate Zn to meet the demand for the battery cycle. 3) The flexible ZAB requires the anode to be bendable, foldable, twistable, compressible, and stretchable while maintaining regular operations.

**Figure 4:**
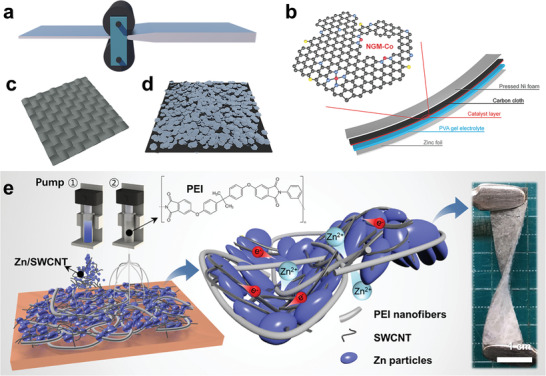
Schematic illustration of various methods to realize the flexibility of Zn metal anode. a) Improves anode flexibility by thinning Zn metal sheet. b) Schematic illustration of the flexible solid ZABs based on thin Zn foil anode and Co/N/O tri‐doped cathode. Reproduced with permission.^[^ ^146^ ^]^ Copyright 2017, Wiley‐VCH. c) Replaces the Zn metal plate with Zn fiber to realize the flexibility of anode. d) Use electrodeposition or coating to obtain a thin layer of Zn to achieve cathode flexibility. e) Schematic diagram of using Zn powder and CNT to construct a flexible anode by electrostatic spraying, and the photograph of the flexible anode. Reproduced with permission.^[^ ^130^ ^]^ Copyright 2018, American Chemical Society.

Generally speaking, Zn metal plates can be directly used as anodes of ZABs.^[^ ^62^, ^173^ ^]^ The untreated Zn metal anodes are usually thick (several millimeters) and prone to mechanical performance degradation or even fracture due to metal fatigue during the deformation process, resulting in undesired consequences for the battery. Therefore, the direct use of Zn foil as a flexible anode usually suffers from the poor mechanical properties caused by an excessively thick metal foil. There are many forms of Zn metal to achieve flexible anodes, as summarized in Table 2. The strain of a specific material is a constant, and the strain formula of film‐like electrodes is as follows

(1) $$\varepsilon =\left(\frac{d_{f}+d_{s}}{R}\right)\frac{\left(1+2\eta +\chi \eta^{2}\right)}{\left(1+\eta \right)\left(1+\chi \right)}$$

where *d*_f_, *d*_s_ is the thickness of the coated film and substrate, *R* corresponds to the radius of curvature of the bend and $\eta =\frac{d_{f}}{d_{s}}$ and $\chi =\frac{Y_{f}}{Y_{s}}$ (*Y*_f_, *Y*_s_ are Young's moduli of the film and substrate, respectively). From the formula, we can find that the strain of the film electrodes is positively correlated with the thickness, so reducing the thickness can effectively reduce the material strain.^[^ ^174^ ^]^ Therefore, a thinner Zn metal sheet can be obtained by mechanical rolling, which brings greater flexibility (**Figure** **4**a). Zhang and co‐workers prepared flexible ZABs using the Zn foil with a thickness of 0.1 mm as the anode (Figure 4b), which was more tightly contacted with the solid‐state electrolyte and the Co/N/O triple‐doped graphene catalyst for the construction of flexible ZABs.^[^ ^146^ ^]^ This battery achieved an open circuit point of 1.44 V and a discharge voltage of 1.19 V at a current density of 1 mA cm^−2^. It maintained a stable cycle under the condition of multiple bends at multiple angles. The above series of work proves the feasibility of using a thinner Zn foil, which could meet the deformation requirements with low utilization of Zn metal simultaneously.

In addition to the thin Zn foil, using Zn metal fiber can also enhance the flexibility of the anode (Figure 4c). The deformability of metal fibers is usually better.^[^ ^175^, ^176^, ^177^ ^]^ High Zn loading can be obtained by weaving metal fibers with high charge transfer capabilities. Zhang and co‐workers have achieved Zn metal fiberization by a metal runner, forming by heating and pressing.^[^ ^111^ ^]^ This fibrous solid anode had good electrical conductivity, superior mechanical stability, controllable mass distribution, and suitable porosity. In addition to obtaining flexible Zn anodes through textiles, it can also be prepared by winding the Zn wire into a spring shape. Liu et al. fabricated a fiber structure flexible ZAB, using a Zn spring combined with a small molecule‐based supramolecular‐polymer double‐network hydrogel electrolytes, and the battery could keep cycling under the condition of 600% stretch.^[^ ^176^ ^]^

The flexibility of the anode can also be achieved by depositing Zn metal particles on the surface of the flexible electrode by coating or pre‐electrochemical deposition (Figure 4d), obtaining flexible Zn anodes with a high metal utilization rate.^[^ ^177^, ^178^ ^]^ Lee and co‐workers used polyetherimide (PEI) from electrospinning as the 3D flexible stock.^[^ ^130^ ^]^ By introducing CNTs on the surface, they constructed a conductive network. A flexible Zn anode was then fabricated by electrostatic spraying Zn powder on the 3D networks, as shown in Figure 4e. Combined with the cathode of rambutan‐shaped cobalt tetroxides coating on multi‐walled CNTs, a flexible ZAB was prepared. The battery exhibited stable electrochemical performance and could cycle even under a crumpled state. Zn layer electrochemical deposition on flexible substrates could also form a flexible anode, apart from the coating method. Zhong and co‐workers deposited Zn on the copper foam by controlling the electroplating time and current density.^[^ ^178^ ^]^ The flexible ZAB exhibited excellent flexibility and cycle stability under bending conditions with the obtained foam as the anode.

Beyond the flexibility, ZABs anode still faces a series of challenges, such as the corrosion of the Zn metal, severe hydrogen evolution, and Zn dendrites caused by uneven deposition of Zn ion.^[^ ^173^, ^175^ ^]^ It is difficult for ZABs to achieve long‐term stable cycles without solving these problems. What is worse, most of the methods for achieving flexibility of the Zn anode will significantly increase the surface area of the anode, which will lead to more severe corrosions of the Zn anode, further leading to the decrease of the cycle performance. Therefore, additional attention needs to be paid to metal anodes protection when designing flexible anodes of the ZABs.

### 3.3 Solid‐State Electrolyte

The electrolyte is another essential part of the battery system. Although aqueous electrolytes can provide significant safety properties of ZABs, SSEs are more preferred in flexible ZABs. It can not only meet the requirement of flexibility but also avoid the risk of liquid electrolyte leakage, further increasing battery safety.^[^ ^179^, ^180^ ^]^ Due to the considerable mechanical strength of the SSE, the growth of Zn dendrites will be largely suppressed, reducing the risk of the short circuit.^[^ ^181^ ^]^ The commonly used SSEs in flexible ZABs are mostly in a quasi‐solid‐state, including gel electrolytes and cellulose‐based membrane electrolytes, as shown in Table 2. Other SSEs, such as inorganic solid electrolytes, are rarely used in ZABs systems for the poor flexibility and low charge transfer capability. The chemical stability of SSEs in metal–air batteries also requires additional consideration due to the existence of active oxygen in the cycling environment.^[^ ^182^ ^]^ Therefore, a qualified SSE should process the following characteristics: 1) sufficient flexibility for meeting the deformation requirements of flexible ZABs; 2) high stability, including the chemical stability and the water retention ability; 3) sufficient ions conductivity; 4) considerable mechanical strength for avoiding short circuit caused by Zn dendrites.

**Figure 5:**
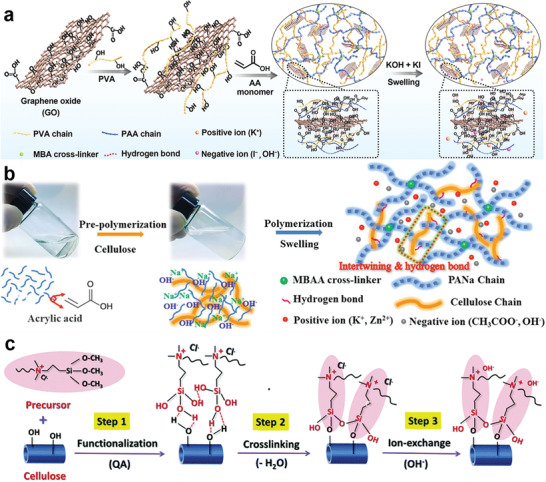
a) Scheme of the fabrication process for KI–PVAA–GO gel electrolyte. Reproduced with permission.^[^ ^183^ ^]^ Copyright 2020, Wiley‐VCH. b) Synthetic procedure of the PANa‐cellulose hydrogel electrolyte using MBAA as crosslinker, acrylate as the main monomer, and cellulose as an enhancer. Reproduced with permission.^[^ ^185^ ^]^ Copyright 2019, Wiley‐VCH. c) Chemical structure evolution of the cellulose nanofiber surface after functionalization, crosslinking, and hydroxide exchange. Reproduced with permission.^[^ ^187^ ^]^ Copyright 2015, Royal Society of Chemistry.

Gel polymer electrolytes can be pretty competitive among SSEs.^[^ ^146^, ^183^ ^]^ The gel electrolyte based on a single polymer usually exhibits poor mechanical properties, which means it is difficult to inhibit the formation of the Zn dendrites. Thus, a large part of the efforts in developing gel electrolytes are devoted to increasing mechanical strength. Meanwhile, due to the semi‐open system of the ZAB, the H_2_O in the electrolyte is easy to lose, the water retention capacity is also vital for the gel electrolyte. Zhong and co‐workers introduced a gel polymer electrolyte by adding poly(acrylic acid) and graphene oxide to the PVA‐based alkaline gel electrolyte (**Figure** **5**a).^[^ ^183^ ^]^ Through multiple crosslinking reactions between the three components, the mechanical strength and the water retention capacity of the original gel electrolyte were improved. Furthermore, the introduction of potassium iodide has successfully changed the OER process and decreased the charge overpotential of the battery. The flexible ZABs using this gel electrolyte can be fabricated into different structures, 1D fiber‐shape, and 2D sandwich structures, exhibiting a low charge potential at 1.69 V with a 200 h stable cycle.

It is also challenging for most gel electrolytes to achieve large‐scale elastic stretching because they will easily lose part of stretchability after adding alkaline solution.^[^ ^184^ ^]^ Zhi and co‐workers have explored the main reasons why polymer hydrogels lose their tensile properties in alkaline environments.^[^ ^185^ ^]^ The superstretchable and normal working dual network gel electrolytes were designed using polyacrylate sodium (PANa) to soften the hydrogel, as shown in Figure 5b. The flexible ZABs based on the gel electrolyte kept cycling normally when stretched to 800%.

It is essential to introduce a gel electrolyte system with a multi‐molecule crosslinking system.^[^ ^185^ ^]^ In addition, introducing hydroxyl groups into the polymer can effectively improve the water retention capacity of the gel electrolyte. Nevertheless, the gel electrolyte is still facing challenges to achieve highly efficient catalysis, such as the poor wettability between the gel electrolyte and the electrodes. Therefore, in addition to mechanical strength, water retention, and deformability, it is still necessary to consider the wettability between the two when designing a new gel electrolyte.

Another type of electrolyte for flexible ZABs that has been studied at length is cellulose membrane solid electrolytes.^[^ ^186^ ^]^ They have improved mechanical properties than polymer gels and can better reduce the volume expansion and the problems caused by dendrite growth.^[^ ^182^ ^]^ At the same time, as a bio‐derived material, cellulose was more environmentally friendly. Chen and co‐workers chose cellulose nanofibers as raw materials, and the nonporous cellulose membrane was obtained through grinding, enzyme treatment, and nano treatment (Figure 5c).^[^ ^187^ ^]^ Through ion exchange and KOH solution infiltration, a cellulose‐based quasi‐solid electrolyte was fabricated. The flexible ZABs with the cellulose‐based quasi‐solid electrolytes also presented good flexibility (it could maintain stable cycling under different bending degrees and light a red LED under bending conditions) and electrochemical performance. Despite the many advantages mentioned above, this type of electrolyte requires the addition of liquid KOH electrolyte, which also brings about the problem of zinc anodes. Moreover, the issue of H_2_O evaporation in the circulation process should be addressed as well.

It should be noted that in ZABs, alkaline electrolytes would be applied in most cases. It would be easily affected by the CO_2_ in the air, resulting in poorer battery performance.^[^ ^188^ ^]^ Meanwhile, the alkaline electrolyte can also cause corrosions of the Zn anode and degradations of the gel electrolyte flexibility. Thus developing nonalkaline gel electrolytes or novel SSEs is also essential for the next‐generation flexible ZABs.

## 4 Structures for Achieving Flexible ZABs

**Figure 6:**
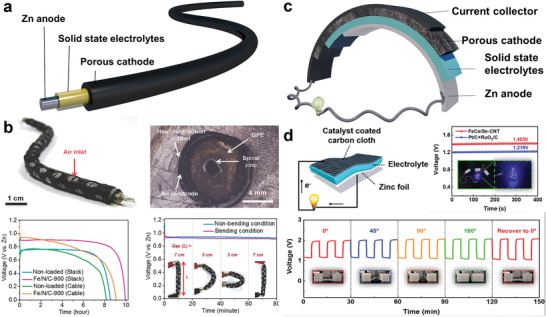
Schematic illustration of two construction methods of flexible ZABs. a) Schematic diagram of fiber‐shape flexible ZABs structure. b) Photograph of fiber‐shape ZABs and optical microscopy for the cross‐sectional image of the fiber‐shape ZABs. Discharge curves of ZABs with and without catalysts. And the curves of fiber‐shape ZABs under different bending angles. Reproduced with permission.^[^ ^190^ ^]^ Copyright 2014, Wiley‐VCH. c) Schematic diagram of sandwich structure flexible ZABs. d) Schematic illustration of 2D sandwich structure Zn–air battery, and the open‐circuit plots of the ZABs based on the catalysts. Cycling stability of solid flexible Zn–air battery with different bending angles. Reproduced with permission.^[^ ^193^ ^]^ Copyright 2021, American Chemical Society.

In addition to flexible electrodes and solid electrolytes, there are also different manifestations in building flexible ZABs. These flexible ZABs with various structures and assembly forms often have different flexibility characteristics. According to the building structures of the cells, the flexible ZABs can be classified into two types.^[^ ^111^, ^183^ ^]^ One is a 1D fiber‐shape battery structure, and the other is 2D sandwich‐structured batteries, as shown in **Figure** **6**.

1D fiber‐shape battery is a common type of flexible battery.^[^ ^185^ ^]^ Usually, the fiber‐shape battery is a twisted‐pair battery, consisting of two fibrous electrodes and solid electrolytes. Since the active cathode material of the ZABs is air, the typical fiber‐shape ZABs adopt coaxial cable configurations. From the inside to the outside are respectively a Zn metal anode, a solid electrolyte layer, and a porous cathode layer (Figure 6a). The Zn^2+^ comes from the inside Zn anode during the cycle and reacts with the O_2_ reduction intermediates, forming discharge products deposited in the porous cathode. This 1D fiber‐shape battery can be connected in series and parallel by weaving and presents advanced flexibility, achieving bending, twisting, and stretching well.^[^ ^189^ ^]^

Cho and co‐workers designed a 1D concentric shaft type flexible ZAB based on a spring‐like Zn anode, a free‐standing gel polymer electrolyte, and a porous cathode (Figure 6b). This fiber‐shaped battery displayed good flexibility and good electrochemical properties. It kept cycling under the condition of more than 50% of compression.^[^ ^190^ ^]^ Zapien and co‐workers coated the Prussian blue on the CNT paper, then through the simple heat treatment of the obtained materials to get Co–N–C nanomaterials as the flexible cathode.^[^ ^191^ ^]^ A spring‐shaped Zn anode and a dual‐network sodium polyacrylate with cellulose‐based hydrogel electrolyte were prepared to form the 1D ZAB. The battery possessed high flexibility as it could be stretched 500% without damage. It also delivered excellent electrochemical performance, releasing a peak power density of 128 mW cm^−2^.

**Figure 7:**
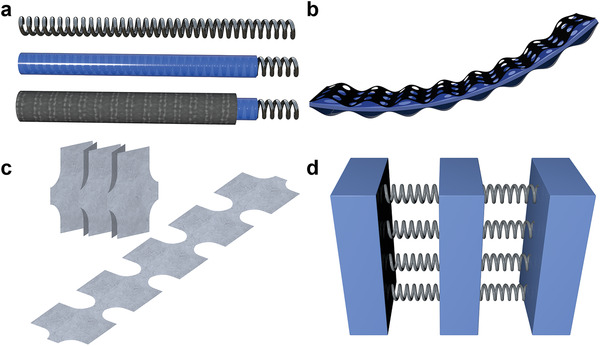
Strategies to achieve large‐scale deformation of flexible new‐air batteries. a) Schematic diagram of realizing battery flexibility through spring structure design. b) Schematic diagram of realizing battery flexibility through wave‐shaped electrode design. c) Schematic diagram of realizing battery flexibility through origami/kirigami structure design. d) Schematic diagram of realizing battery flexibility through island‐bridge design.

Pre‐designing the battery structure can effectively improve the flexibility of 1D fiber‐shape structure batteries, such as purposing the battery into a spring shape, as shown in **Figure** **7**a. It has very large deformability, which can be stretched, compressed, bent, etc.^[^ ^185^ ^]^ In some cases, it can be stretched several times or compressed 80% while working.^[^ ^187^, ^192^ ^]^ In addition, by weaving the fiber‐shape batteries, flexible packages of a set of batteries can be connected. It is even possible to directly assemble the fiber‐shape ZABs into the flexible device by weaving to obtain the overall flexibility of the devices.

The 2D sandwich structure is one of the earliest and most used battery designs among the flexible ZABs.^[^ ^104^, ^146^ ^]^ This kind of ZAB is built up by a Zn anode, an SSE, and a porous cathode, layer‐by‐layer stacking together between two current collectors (Figure 6c).^[^ ^108^ ^]^ Commonly used current collectors include stainless steel, copper foil, and carbon cloth.^[^ ^146^, ^185^ ^]^ Zhang and co‐workers grew a porphyrin‐based COF on the CNT framework to form an independent and flexible film as the cathode. Combined with a solid electrolyte and a Zn metal anode, a sandwich structure ZABs was finally obtained.^[^ ^104^ ^]^ This battery exhibited an overpotential at 0.71 V and kept stably cycling for 200 cycles. At the same time, it lighted up red LED bulbs under different curvatures. Huang and co‐workers synthesized an ultra‐stable FeCo bifunctional oxygen electrocatalyst on Se‐doped CNT (FeCo/Se‐CNT) through a gravity‐guided CVD strategy, as shown in Figure 6d.^[^ ^193^ ^]^ The catalyst was combined with a gel electrolyte and a Zn sheet to form a 2D sandwich structure ZABs. The battery showed no apparent performance degradation during the bending process. Compared with the 1D structure, the combination of 2D flexible ZABs is relatively simple: they can be connected either in parallel or in series by stacking or end to end.

**Figure 8:**
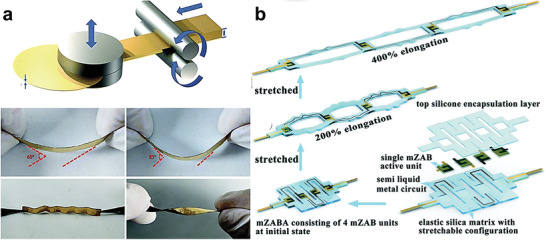
a) Scheme of the flexible ZABs and the demonstration of Au foil manufacturing process. Reproduced with permission.^[^ ^196^ ^]^ Copyright 2020, Royal Society of Chemistry. b) Schematic diagram for the fabrication of the stretchable ZABs is based on a bridge‐island design. Reproduced with permission.^[^ ^199^ ^]^ Copyright 2021, Royal Society of Chemistry.

Different 2D designs need to be figured out according to diverse deformation requirements.^[^ ^194^ ^]^ Among them, the wave or pre‐strained structures are typical examples of significant deformation needs.^[^ ^195^ ^]^ The electrode material is deposited on a pre‐strained elastic substrate, then releases strain to obtain an electrode with a wave structure, as shown in Figure 7b. Due to the pre‐deformation, the finally prepared wave‐shaped electrode can offer very excellent flexibility. When combined with a flexible solid electrolyte and a metal anode, the flexible ZABs can even cycle during stretching. Huang and co‐workers obtained a very thin gold sheet through the rolling method, as shown in **Figure** **8**a.^[^ ^196^ ^]^ Then the obtained gold sheet was attached to the CNT paper. A wave‐shaped flexible ZAB was finally obtained by combining the Au/CNT, PANa gel electrolyte, and a flexible Zn anode. The battery exhibited 300% tensile and 80% compressive during cycling.

Besides the wavy structure, the origami/kirigami structure is also an often‐used design that can achieve large‐scale deformation (Figure 7c).^[^ ^197^ ^]^ This type of structure pre‐folds the flat battery repeatedly to achieve the purpose of pre‐deformation. Such structure can be opened out directly to achieve deformation. However, unlike the wavy structure, as the strain of the origami structure is concentrated in specific areas, particular designs are required to avoid breakage. For example, suitable materials are preferred to strengthen particular regions.

Another unique battery design, the island‐bridge design, has recently emerged.^[^ ^198^ ^]^ This structure has two parts, the rigid part (which could be called an island) and the elastic part (which could be called a bridge.) (Figure 7d). The deformation process of the battery is achieved by the stretching and shrinking of the bridge. A typical bridge is composed of long thin metal wires or liquid metals. Wu and co‐workers used interdigital carbon cloth as the cathode, the interdigital Zn as the anode, and the liquid gallium indium alloy as the flexible bridge (Figure 8b).^[^ ^199^ ^]^ The obtained island‐bridge structural ZABs had a stable electrochemical output even when stretched to 400%.

When a set of batteries are connected, island‐bridge structures or origami/kirigami structures can be applied, but it should be noted that introducing components for better deformation would lead to system energy density loss. Therefore, when designing a flexible ZABs pack, it is essential to consider the balance between flexibility and energy density.

## 5 Conclusion and Prospects

In general, compared with other energy storage systems, ZABs are considered a promising energy supply for flexible devices due to their high safety, considerably high energy density, and structural designable ability. For flexible battery designing, in addition to the consideration of electrochemical properties, structural engineering is also essential to achieve superior performance of the batteries. The construction strategies of flexible electrodes, the design of SSEs, and different deformable battery structures have been introduced and discussed in this review. Although many achievements have been witnessed of flexible ZABs, some challenges should not be neglected for their practical applications.

1. Aligning test standards of flexibility. The degree of flexibility of a battery is critical. However, current research mainly focuses on electrochemical performance and there lack of standards for the flexibility tests of batteries. Universal and standard testing methods need to be established and aligned.
2. Higher Energy density. There are inevitable loose structures or redundant parts in the design of the flexible ZABs to gain deformability, which will significantly affect the overall energy density. Therefore, it is necessary to optimize the flexible ZABs and balance flexibility and energy density.
3. Air cathode design. In addition to the energy density, attention needs to be paid to the low power density of the system, which is mainly caused by slow mass transfer and high reaction energy barrier. It should be noted that more factors need to be considered for flexible ZABs compared to traditional LIBs or other technological matured closed batteries systems. Different factors, such as the catalyst, mass transfer, electrical conductivity, air cathode structure and deformability, cannot be neglected when designing. Besides, the impact of CO_2_ from the air and the issue of water evaporation need to be also considered.
4. Flexible anode protection. Attention needs to be paid to undesired Zn corrosion or severe dendrites growth during cycling, which will affect the life of all Zn‐based batteries. Although the existed flexible anode can achieve basic flexibility, novel deformable designs for Zn anode are expected.
5. Developments of SSEs. Currently, the electrolyte of flexible ZABs is mostly quasi‐solid, and adding the liquid electrolyte will lead to severe water evaporation. Meanwhile, the addition of an alkaline electrolyte also makes the battery sensitive to CO_2_. It is crucial to develop nonalkaline gel electrolytes and other kinds of all‐solid‐state electrolytes. In addition, it is also vital to design an SSEs that can meet large‐scale deformations.
6. Flexible structure design. The flexibility and the energy supply requirements should be considered simultaneously, and it needs customized designs according to the various service occasions and requirements. When necessary, flexible battery packs can be achieved by combining various structural designs.

The flexible ZABs can be promising among the candidates, aiming at high energy density, increased safety, and considerable deformability. This review will be helpful to the researchers studying in this area, and more progress will be achieved to address the issues hindering the practical applications of flexible energy storage devices.

## Conflict of Interest

The authors declare no conflict of interest.
